# Cost-effectiveness of direct oral anticoagulants versus vitamin K antagonist in atrial fibrillation: A study protocol using Real-World Data from Catalonia (FantasTIC Study)

**DOI:** 10.1097/MD.0000000000022054

**Published:** 2020-09-04

**Authors:** Zojaina Hernández Rojas, Maria Rosa Dalmau Llorca, Carina Aguilar Martín, Alessandra Queiroga Gonçalves, Marc Casajuana, José Fernández-Sáez, Dolores Rodríguez Cumplido, Emma Forcadell Drago, Noèlia Carrasco-Querol, Josep Maria Pepió Vilaubí, Josep M. Alegret

**Affiliations:** a Equip d’Atenció Primària Tortosa Est, Institut Català de la Salut, Tortosa, Tarragona, Spain; b Unitat de Suport a la Recerca Terres de l’Ebre, Fundació Institut Universitari per a la Recerca a l’Atenció Primària de Salut Jordi Gol i Gurina (IDIAPJGol), Tortosa, Tarragona, Spain; c Universitat Rovira i Virgili, Tarragona, Spain; d GAVINA Research Grup; e Unitat d’Avaluació, Direcció d’Atenció Primària Terres de l’Ebre, Institut Català de la Salut, Tortosa, Tarragona, Spain; f Unitat Docent de Medicina de Familia i Comunitària, Tortosa-Terres de l’Ebre, Institut Català de la Salut, Tortosa, Tarragona, Spain; g Fundació Institut Universitari per a la recerca a l’Atenció Primària de Salut Jordi Gol i Gurina (IDIAPJGol), Barcelona, Spain; h Universitat Autònoma de Barcelona, Bellaterra (Cerdanyola del Vallès), Spain; i Unitat de Recerca, Gerència Territorial Terres de l’Ebre, Institut Català de la Salut, Tortosa, Tarragona, Spain; j Facultat d’Enfermeria, Campus Terres de l’Ebre, Universitat Rovira i Virgili, Tortosa, Tarragona, Spain; k Hospital Universitari de Bellvitge, Institut Català de la Salut, Barcelona, Spain; l Equip d’Atenció Primària Tortosa Oest, Institut Català de la Salut, Tortosa, Tarragona, Spain; m Grup de Recerca Cardiovascular, Departament de Cardiologia, Hospital Universitari de Sant Joan, Institut de Investigació Sanitaria Pere Virgili (IISPV), Reus, Spain; n Departament de Medicina i Cirurgia, Universitat Rovira i Virgili, Reus, Spain.

**Keywords:** atrial fibrillation, cost effectiveness, primary health care, anticoagulants, oral drug administration

## Abstract

**Background:**

Anticoagulant therapy is used for stroke prevention and proved to be effective and safe in the long term. The study aims to analyse the cost-effectiveness relationship of using of direct-acting oral anticoagulants vs vitamin K antagonists to prevent ischaemic stroke in patients with nonvalvular atrial fibrillation, including all the active ingredients marketed in Spain, prescribed for 2 years in the Primary Care service of the *Institut Català de la Salut*.

**Methods:**

Population-based cohort study, in which the cost of the 2 treatment groups will be evaluated. Direct costs (pharmacy, primary care, emergency and hospitalization) and indirect costs (lost productivity) will be included from a social perspective. Effectiveness (assessed as the occurrence of a health event, the 1 of primary interest being stroke) will be determined, with a 2-year time horizon and a 3% discount rate. The average cost of the 2 groups of drugs will be compared using a regression model to determine the factors with the greatest influence on determining costs. We will carry out a univariate (‘one-way’) deterministic sensitivity analysis.

**Discussion:**

We hope to provide relevant information about direct and indirect costs of oral anticoagulants, which, together with aspects of effectiveness and safety, could help shape the consensual decision-making of evaluating bodies.

## 1 Introduction

Atrial fibrillation (AF) is the most common arrhythmia in western countries and is associated with high morbidity and mortality, thereby constituting a major public health problem.^[1,2]^ It is 1 of the most frequent causes of ischaemic stroke.^[3,4]^ Anticoagulant therapy is used for stroke prevention, for which there are 2 main groups of drugs —vitamin K antagonists (VKAs) and direct-acting oral anticoagulants (DOACs)— which have both proved effective and safe in the long term.^[5]^

VKAs have been widely used for a long time and their efficacy in preventing strokes and systemic embolism in patients with nonvalvular atrial fibrillation (NVAF) is well established.^[6]^ However, most of the studies have involved warfarin, and the results have been generalized to all VKAs.^[7]^ The price of these drugs is low (compared with that of DOACs),^[8]^ although they generate direct and indirect costs. Furthermore, they have limitations such as interindividual and intraindividual variability of response, a slow onset of action, a narrow therapeutic margin, a need for dose-adjustment through periodic controls of the international normalized ratio, and interactions with some foods and medications.^[9–11]^

In recent years, DOACs have emerged that have more predictable anticoagulant effects and fewer interactions with other drugs, and that allow a fixed dosage regimen, without the need for monitoring.^[12]^ Overall, DOACs present a favorable risk prevention profile, with significantly lower levels of strokes, intracranial haemorrhages and mortality compared with VKAs, but they have a higher risk of gastrointestinal bleeding^[7]^ and are expensive.^[8]^ It is also important to take into account the adjustment of the dosage according to age, weight, and renal function, in addition to monitoring compliance, because highly variable therapeutic adherence rates have been noted.^[7,13]^

The European and American guidelines recommend the use of any DOACs rather than VKAs because of their net clinical benefit, except in selected cases.^[14–16]^ However, the use of DOACs in clinical practice is determined by the recommendations of the health authorities of each country. In Spain, VKAs are recommended as the first choice, with DOACs being used when specifically recommended,^[17]^ although currently it has been observed that more than 80% of physicians are starting treatments with DOACs .^[18]^

Previous studies found that DOACs tend to be more cost-effective than VKAs for the treatment of AF.^[19–23]^ One of these studies suggest that rivaroxaban is cost-effective compared to VKA, although the type of AVK and the dose thereof are not specified.^[23]^ A comparative study of the cost-utility of the first 3 DOACs to be marketed in Spain (apixaban, dabigatran and rivaroxaban),^[24]^ found dabigatran to be the most cost-useful. ^[25]^ However, the most recent study carried out in Spain does not show significant differences between dabigatran and AVKs.^[26]^ A recent study of edoxaban in Spain concluded that it is a cost-effective alternative for this indication.^[27]^ However, we did not find any cost-effectiveness studies that included all the anticoagulants currently available on the market, with a population base (without economic models) and real world data (RWD).

The main objective of the study will be to analyse the cost-effectiveness of using DOACs in comparison with VKAs to prevent ischaemic stroke in patients with NVAF, including all the active ingredients marketed in Spain prescribed for 2 years in the Primary Care (PC) service of the *Institut Català de la Salut* (ICS). As secondary objectives, we intend to:

(1) determine the appearance of a health event, according to the type of anticoagulation used (VKA or DOAC), and the factors related to this;
(2) evaluate the cost in patients who are poorly controlled by VKAs and under-treated with DOACs; and
(3) analyse the factors associated with increased costs.

The present study protocol, named FantasTIC (Non-valvular atrial fibrillation and treatment, health assessment, Information and Communication Technologies) study has been designed to address these aspects.

## 2 Methodology

### 2.1 Design and study period

**Figure 1:**
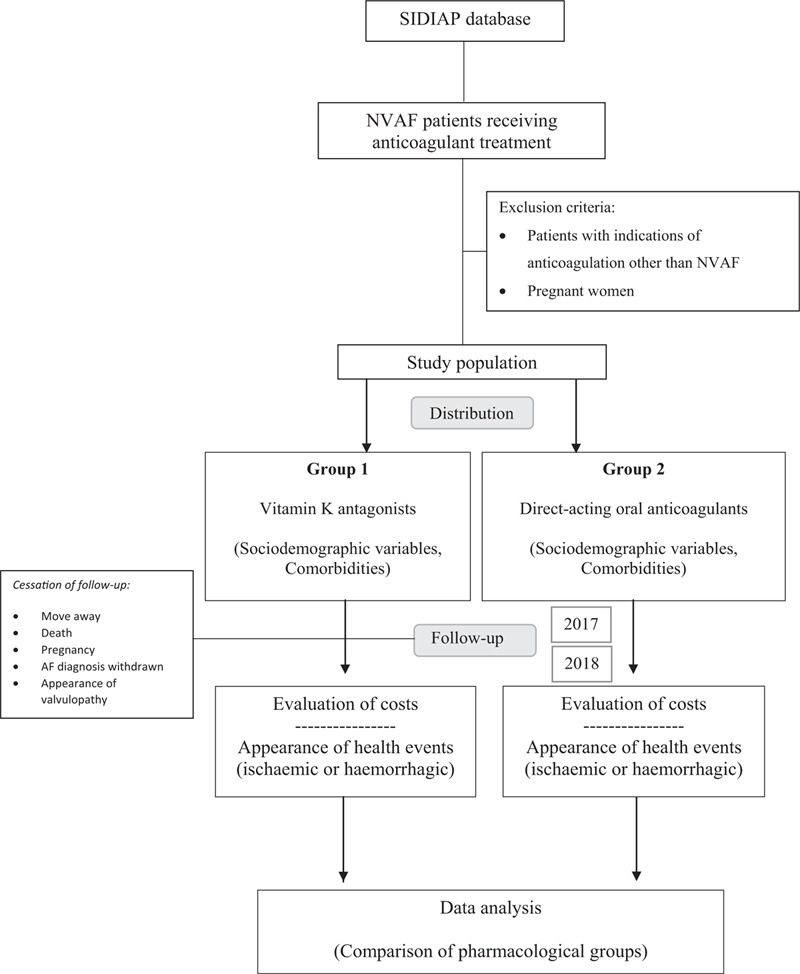
Flow diagram.

This is a population-based cohort study designed to evaluate and compare the cost-effectiveness of the 2 types of anticoagulant treatment (VKA and DOAC) in patients with NVAF, with a time horizon of 2 years (from 1 January 2017 until 31 December 2018). The flow diagram of the study is presented in Figure 1.

### 2.2 Study population

Our study population will be that treated by the PC service of the ICS. The Autonomous Community of Catalonia has a health service whose main provider is the ICS, in which PC is organized into teams made up of family doctors, nurses, paediatricians, social workers, dentists and support staff. The ICS manages, among others, 287 PC centres (PCCs) to which 5,564,292 citizens are assigned (approximately 80% of the Catalan population).^[28]^

As established by the *Sistema de Información para el Desarrollo de la Investigación en Atención Primaria* (SIDIAP; Information System for the Development of Research in PC), during the year 2017, 126,702 cases of NVAF had been registered in the ICS, 61,002 of which were receiving anticoagulant treatment (41,430 VKA and 19,548 DOAC).

Inclusion criteria:

(1) Patient with a diagnosis of NVAF at least 1 year old.
(2) Patient undergoing treatment with VKA or DOAC.

Exclusion criteria:

(1) Patient with indications for anticoagulation other than NVAF.
(2) Pregnancy.

Data collection will end on 31 December 2018 or sooner if any of the following occurs:

(1) Anticoagulation prescription suspended for more than 180 days.
(2) Patient moves to another Autonomous Community.
(3) Patient becomes pregnant.
(4) Withdrawal of the diagnosis of NVAF.
(5) Appearance of a diagnosis of valvular AF.
(6) Death of the patient.

### 2.3 Data source

At ICS, more than 9175 professionals working in PC use the same computerized medical history program, called eCAP. eCAP data will be obtained through SIDIAP, which is a unique database, previously validated, and highly representative of the Catalan population.^[29,30]^

In this way, SIDIAP provides, from each of the 5.8 million citizens assigned to the various PCCs of the ICS, information linked to a unique, anonymised identifier. The following information will be obtained from all NVAF patients receiving anticoagulants on January 1, 2017:

(1) data from the eCAP program: demographic data, PC visits, health events, clinical variables, referrals, deaths, prescriptions and sick leave;
(2) laboratory results: these will be extracted directly from the laboratory database, rather than depending on manual records, thereby guaranteeing data quality;
(3) medication dispensed by pharmacy offices: this information will be obtained directly from those offices;
(4) other, external sources of information: (4.1.) *Conjunt Minim Bàsic de Dades* (CMBD), which is a population registry that collects ICD-9 pathology data linked to hospitalization at all of the Hospitals in Catalonia,^[31]^ (4.2.) Mortality: data supplied by the Department of Health, including cause and date of all deaths of residents of Catalonia.^[28]^

### 2.4 Variables

The variables considered in the study will be divided into 5 groups: cost, follow-up, effectiveness, pharmacy, and those corresponding to personal history (summarised in Table 1).

**Table 1:**
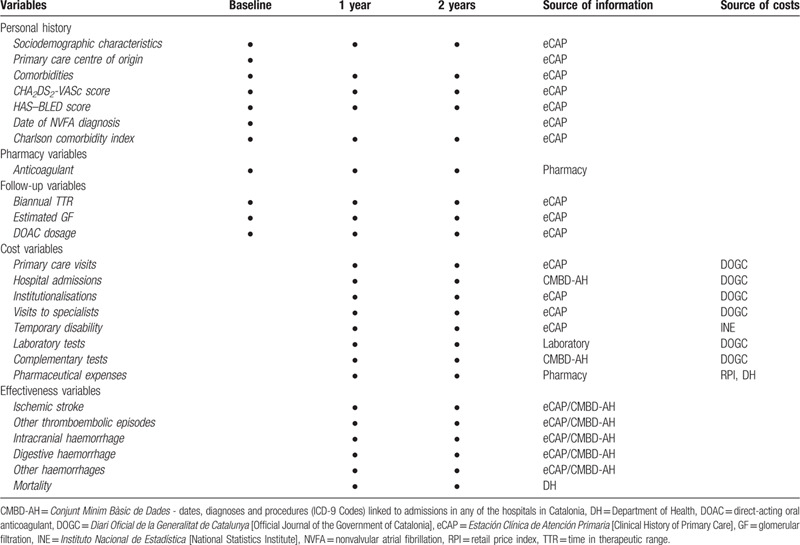
Variables, sampling period, and source of information.

All oral anticoagulants that are marketed in Spain will be included in the study:

(1) VKAs: Acenocoumarol and warfarin.
(2) DOACs: Apixaban, dabigatran, edoxaban and rivaroxaban.

### 2.5 Cost estimation

Cost estimates will take into account direct expenses (from pharmacy, PC, emergencies and hospitalisation) and indirect expenses (loss of productivity), from a social perspective, in patients diagnosed with NVAF and treatment with oral anticoagulants. Two groups will be established: patients receiving VKAs and those receiving DOACs. We will use a 3% discount rate, which will be applied in the second year.

We will use the charges set out in the *Diari Oficial de la Generalitat de Catalunya* (DOGC)^[32]^ closest to the date of analysis (2014) to estimate the costs. The euro will be used as the reference currency (which will not require conversion). Prices will be adjusted to those of 2018 to take into account inflation in the health sector.^[33]^ The costs of hospital admissions will be obtained from the *Grups Relacionats amb el Diagnòstic* (GRD) patient classification system, published in the DOGC.^[34,35]^ For drug prices, the retail price taken from pharmacy billing data will be used. The final costs of the 2 study groups (patients treated with VKAs and those treated with DOACs) will be calculated by quantifying the resources consumed by each individual with respect to each variable and then multiplying by the cost of each resource. This procedure will yield the total cost of the resource per individual, as well as the total cost for each study group.

### 2.6 Measurement of effectiveness

The primary health event in this project will be “ischaemic stroke” and the indicator of cost-effectiveness will be “avoided strokes”. To estimate avoided strokes we will determine the total economic cost for the 2 pharmacological groups, as well as the number of strokes registered during the 2 years of the study period. Finally, we will compare the groups and thereby determine the economic cost of avoiding a stroke.

Secondarily, we will determine the appearance of the following health events during the 2 years of the study:

- Ischaemic events:
  (1) Ischemic stroke.
  (2) Other thromboembolic events (transient ischaemic attack (TIA), angina, acute myocardial infarction, and peripheral embolism).
  • Haemorrhagic events:
  (1) Intracranial haemorrhage.
  (2) Gastrointestinal bleeding.
  (3) Other haemorrhages.
  • All-cause mortality.

### 2.7 Economic analysis

The cost of DOACs will be compared with that of VKAs, following the methodological guidelines for this type of study proposed by the International Society for Pharmacoeconomics and Outcomes Research (ISPOR)^[36]^ and the International Network of Agencies for Health Technology Assessment (INAHTA).^[37]^

After comparing the average cost of both groups of drugs and estimating their confidence intervals, a regression model will be developed to determine which factors may have the most substantial influence on determining costs. A generalized linear model (GLM) will be derived, which, given the asymmetry of the cost variable, will be based on the gamma family of distributions, with a log link function,^[38]^ because these give a best fit to the data when estimating the impact of the factors associated with the composition of patient costs.

### 2.8 Sensitivity analysis

A univariate (1-way) deterministic sensitivity analysis will be carried out, in the items which the greatest impact on the composition of the cost, or in which there may be a uncertainty with the real price.^[39]^ This will allow us to test the robustness of our results.

## 3 Ethics and dissemination

This study will be carried out in accordance with the norms and principles of the Declaration of Helsinki. The protocol was approved by the Clinical Research Ethics Committee of the *Fundació Institut Universitari per a la recerca a l’ Atenció Primària de salut Jordi Gol i Gurina (IDIAPJGol)* on May 30, 2018 with code P18/080, and has also been authorized by the Primary Healthcare Directorate of the ICS. The SIDIAP database provides anonymised data, identified with an internal code, which makes it impossible, even for the research team, to identify any subject. For the same reason, informed consent will not be obtained. By these means, the confidentiality of the data of the individuals included in the study will be guaranteed, in accordance with the Organic Law on Protection of Personal Data (03/2018 of December 5, and in accordance with the provisions of the Regulation 2016/679 of the European Parliament and of the Council of 27 April 2016 on data protection, and all applicable national regulations. If any modification is required, it will be presented to the IDIAPJGol Clinical Research Ethics Committee for approval.

The results and conclusions of this study will be disseminated in Spain, as well as in international scientific and professional forums, with the results published in indexed scientific journals.

## 4 Discussion

The aim of the present study is to carry out an economic evaluation of all the anticoagulants indicated for the prevention of thromboembolic complications in patients with NVAF.

VKAs are the most used anticoagulants worldwide.^[40]^ However, the administration criteria differs in each country. For example, while in Continental Europe, the VKA most widely used is the acenocoumarol, in the United States and the rest of Europe, it is the warfarin.^[41]^ Several studies have been carried out with warfarin. ^[15,16,19,20,25,42]^ Though, there is scarce literature on acenocoumarol, ^[21,22,24,27]^ which is the most popular VKA in Spain. In the clinical practice, the results of the studies including warfarin are assumed to be generalizable.^[7]^ Nevertheless, and from the best of our knowledge, there is no evidence to support this assumption.

The main strength of our study is the analysis of all marketed anticoagulants from Spain, which will provide strong evidence about their differences, including both VKAs (warfarin and acenocoumarol). Another strength includes the quantification of indirect costs, such as the loss of productivity, associated with the 2 types of treatments. Since it is a population-based study, with RWD, more accurate and representative information will be available for health decision-makers.^[43,44]^ Moreover, most of the published research on this topic is based on hospital data, whereas our project will include variables and expenses related to PC settings, where most VKA controls are performed in our milieu.

### 4.1 Limitations

Our study has certain limitations. First, the database includes data that are dependent on the professional registry, which means that there may be some under-registration of some variables of interest. In addition, there are expenses that we cannot determine, such as those of medical transport or the journeys undertaken by patients themselves, which could therefore lead to the costs of VKA being underestimated. Also, we will not be able to include intangible costs (as patient suffering due to complications or caregiver overload) and operating costs, (such as overheads: water, furniture, building maintenance and input costs). However, these limitations should affect both groups of anticoagulants equally, so it is reasonable to assume that they will not have a significant impact on the result. Finally, a time horizon of 2 years could be too short a time for a health event to occur.

### 4.2 Future directions

The evaluation of drugs with real-world data, based on cost-effectiveness analyses, provides a valuable insight for other European countries, where the conditions of use of these drugs are usually similar.

This study could be of interest throughout Spain, as well as to other countries with similar guidelines for treating NVAF, as it will provide relevant information about direct and indirect costs, which, together with aspects of effectiveness and safety, could help shape the consensual decision-making of evaluating bodies, and thereby provide a stimulus to update current consensus documents. On the other hand, we consider that it would be interesting for future research to include the opinions of patients about their preferences for 1 type anticoagulant over another, since this is a factor that could influence adherence to treatment and, indirectly, its effectiveness and economic cost.

## Acknowledgments

The authors acknowledge *IDIAPJGol* for having awarded the database scholarships and for enabling the intensification of the research activity, both of which are essential for the successful execution of the present study. We also thank the *Fundació Dr. Ferran* for the scholarship and their support for the *Generalitat de Catalunya's Industrial Doctorate* training programme. We greatly appreciate the valuable support of Dr Maria Ferré and Nurse Nuria Beguer.

## Author contributions

**Conceptualization:** Zojaina Hernández Rojas, Maria Rosa Dalmau Llorca, Carina Aguilar Martín.

**Data curation:** Carina Aguilar Martín, Marc Casajuana, José Fernández-Sáez.

**Funding acquisition:** Zojaina Hernández Rojas, Maria Rosa Dalmau Llorca.

**Formal analysis:** Marc Casajuana, José Fernández-Sáez.

**Investigation:** Zojaina Hernández Rojas, Maria Rosa Dalmau Llorca, Carina Aguilar Martín, Marc Casajuana, Dolores Rodríguez Cumplido, Emma Forcadell Drago, Noèlia Carrasco-Querol, Josep Maria Pepio Vilaubí, Alessandra Queiroga Gonçalves, Josep M Alegret.

**Methodology:** Carina Aguilar Martín, Marc Casajuana, Alessandra Queiroga Gonçalves, Dolores Rodríguez.

**Project administration:** Zojaina Hernández Rojas, Maria Rosa Dalmau Llorca.

**Supervision:** Maria Rosa Dalmau Llorca, Josep M Alegret.

**Validation:** José Fernández-Sáez.

**Visualization:** Dolores Rodríguez Cumplido, Emma Forcadell Drago.

**Writing – original draft:** Zojaina Hernández Rojas.

**Writing – review and editing:** Maria Rosa Dalmau Llorca, Carina Aguilar Martín, Marc Casajuana, Alessandra Queiroga Gonçalves, José FernándezSáez, Dolores Rodríguez Cumplido, Emma Forcadell Drago, Noèlia Carrasco-Querol, Josep Maria Pepio Vilaubí, Josep M Alegret.
